# Identification and validation of key biomarkers of the glycolysis-ketone body metabolism in heart failure based on multi-omics and machine learning

**DOI:** 10.3389/fcvm.2025.1672513

**Published:** 2025-11-27

**Authors:** Na Xiao, Jing Liu, Zhe Chen, Xiaoyong Geng

**Affiliations:** Cardiology, Hebei Medical University Third Hospital, Shijiazhuang, Hebei, China

**Keywords:** heart failure, glycolysis, ketone body metabolism, immune infiltration, machine learning

## Abstract

**Background:**

Metabolic remodeling, particularly involving glycolysis and ketone body metabolism, is a hallmark of heart failure (HF) pathophysiology. However, the regulatory network linking energy metabolism with immune dysregulation remains poorly understood.

**Objectives:**

This study aimed to identify and validate key biomarkers within the glycolysis-ketone body metabolism axis that contribute to the progression of HF, and to explore their association with immune microenvironment alterations.

**Methods:**

Transcriptomic data from HF patients were integrated with glycolysis and ketone metabolism gene sets. Differentially expressed genes (DEGs) were identified and analyzed through Weighted Gene Co-expression Network Analysis (WGCNA). Candidate genes were refined using machine learning algorithms (LASSO regression and Boruta), with functional enrichment assessed via Gene Set Enrichment Analysis (GSEA). Immune infiltration was profiled using ssGSEA, and regulatory networks were constructed by integrating miRNA and transcription factor predictions. Experimental validation was conducted in a murine myocardial infarction model using qPCR and cardiac ultrasound imaging.

**Results:**

Five candidate genes related to glycolysis and ketone metabolism were identified, among which TIMP1 emerged as the key hub gene. TIMP1 expression was significantly elevated in HF and correlated with enriched pathways including inflammatory signaling and mitochondrial dysfunction. Immune profiling revealed that TIMP1 positively associated with the infiltration of activated CD8⁺ T cells and dendritic cells, potentially mediated by chemokines such as CCL2. Regulatory network analysis suggested that upstream transcription factors and miRNAs may contribute to TIMP1 overexpression. Animal model validation confirmed the upregulation of TIMP1 and other core genes, supporting its central role in HF progression.

**Conclusion:**

This study identifies TIMP1 as a central regulator linking glycolysis-ketone metabolic imbalance with immune microenvironment dysregulation in heart failure. These findings offer new mechanistic insights and propose TIMP1 as a potential diagnostic biomarker and therapeutic target in HF.

## 1 Introduction

Heart failure (HF) is a clinical syndrome characterized by impaired ventricular filling or ejection capacity resulting from structural or functional cardiac abnormalities. Its hallmark manifestations include exertional dyspnea, fatigue, fluid retention, and reduced exercise tolerance (1, 2). It is estimated that nearly 64.9 million people worldwide were affected by HF in 2023. Despite significant advancements in pharmacological therapies (such as ARNI and SGLT2 inhibitors) (3, 4), device-based interventions (5), and comprehensive management strategies (6), the long-term prognosis for HF patients remains poor, with a 5-year mortality rate still approximating 50% (7, 8). The fundamental pathological essence of HF involves activation of neurohormonal and inflammatory responses triggered by an initial cardiac injury. This leads to myocardial remodeling, ultimately resulting in progressive deterioration of cardiac function and systemic circulatory dysfunction (9, 10).

Energy metabolism dysregulation represents a core driver in HF progression (11). Its fundamental nature lies in the shift of cardiomyocytes—triggered by ischemia, hypoxia, or dysregulated gene expression—from highly efficient fatty acid oxidation towards inefficient glucose glycolysis for energy production. This shift is accompanied by mitochondrial dysfunction and reduced ATP synthesis, ultimately culminating in a state of cardiac “energy starvation" (12). Glycolysis and ketone body metabolism collectively constitute the metabolic reprogramming characteristic of HF (13, 14). While glycolytic activation provides short-term compensation for ATP deficits, it is inefficient and leads to lactate accumulation (15). The compensatory ketone body metabolism involves the transport of ketone bodies into mitochondria via SLC16A1 for oxidation, thereby bypassing impaired *β*-oxidation and pyruvate dehydrogenase pathways (16). Long-term imbalance between glycolysis and ketone metabolism exacerbates cardiomyocyte apoptosis and fibrosis (17). However, the nature of the interaction (synergistic or antagonistic) between glycolysis and ketone metabolism remains unclear. Key common regulatory nodes have yet to be elucidated. Furthermore, fragmented research focusing on single metabolic markers (e.g., lactate, *β*-hydroxybutyrate) fails to capture the heterogeneous nature of HF. There is a critical lack of comprehensive multi-dimensional integrated diagnostic models, and an urgent need exists for precise therapeutic targets based on metabolic subtype classification. Therefore, this study aims to unveil the key hub genes within the glycolysis-ketone metabolism axis in heart failure and delineate their immune-metabolic interaction networks. Our objective is to provide novel targets for developing precision intervention strategies based on the regulation of metabolic reprogramming.

Building upon this foundation, this study integrated bioinformatics approaches. Transcriptomic data from heart failure patients and metabolic pathway gene sets were acquired by mining public databases (GEO, MSigDB). Differentially expressed genes (DEGs) were identified using the limma package, and Weighted Gene Co-expression Network Analysis (WGCNA) was employed to screen key modules and candidate genes associated with glycolysis/ketone metabolism. Subsequently, machine learning algorithms (LASSO regression, Boruta feature selection) were utilized to refine the core targets, identifying TIMP1 as a central player. In-depth functional characterization of TIMP1 was performed using single-gene Gene Set Enrichment Analysis (GSEA). Furthermore, its role in the immune microenvironment was assessed via immune infiltration analysis using single-sample GSEA (ssGSEA), and its upstream regulatory mechanisms were explored through regulatory network prediction (miRNA and Transcription Factor prediction). Finally, *in vivo* expression validation was conducted by establishing a murine myocardial infarction (MI) model. This step completed the closed-loop research strategy, transitioning from computational prediction to experimental verification. This study represents the first report identifying TIMP1 as a hub gene orchestrating the glycolysis-ketone metabolism-immune imbalance axis in heart failure. It provides novel targets and a theoretical foundation for gaining deeper insights into the mechanisms underlying dysregulation of the metabolic-immune microenvironment in HF and for developing targeted intervention strategies.

## 2 Materials and methods

### 2.1 Data collection

Heart failure transcriptomics data were downloaded from the Gene Expression Omnibus (GEO), specifically the GSE5406 dataset, which included 16 control and 194 heart failure samples of left ventricular tissue, sequenced using the GPL96 Illumina Genome Analyzer platform (Homo sapiens). The glycolysis-related gene sets GOBP_GLYCOLYTIC_PROCESS_THROUGH_FRUCTOSE_6_PHOSPHATE and GOBP_GLYCOLYTIC_PROCESS_THROUGH_GLUCOSE_6_PHOSPHATE, as well as the ketone metabolism-related gene set GOBP_CELLULAR_KETONE_METABOLIC_PROCESS, were downloaded from the Molecular Signatures Database (MSigDB) (18).

### 2.2 Identification of differentially expressed genes DEGs

Differentially expressed genes (DEGs) were computationally determined through the limma algorithm in R software. Statistical thresholds for DEG classification were as follows: up-regulated genes: *p* < 0.05 and fold change >1.25; down-regulated genes: *p* < 0.05 and fold change <1/1.25 = 0.8. Visual representations of transcriptional dynamics, including volcano plots and clustered heatmaps, were created via the ggplot2 and ComplexHeatmap packages, respectively. We finally identified 761 DEGs.

### 2.3 Weighted gene co-expression network analysis (WGCNA) and candidate genes selection

We performed WGCNA on the expression matrix using the R package“WGCNA” (19). All samples were hierarchically clustered using Euclidean distance based on gene expression levels to identify and remove outliers. A critical step before network construction is determining the optimal soft thresholding power (*β*) to approximate a scale-free topology. The power parameter *β* strengthens strong correlations and penalizes weak ones by raising Pearson correlation coefficients to the power of *β*. We systematically tested values of *β* from 1 to 20 and evaluated two key metrics: (1) The scale-free topology fit index (R^2^); (2) The mean connectivity of the network. We selected *β* = 7 as it achieved an R^2^ ≥ 0.85 (crossing the red cutoff line) while maintaining relatively low mean connectivity approaching zero, ensuring the network adhered to scale-free properties characteristic of biological systems. Using the chosen soft threshold (*β* = 7), we built an adjacency matrix and then transformed it into a topological overlap matrix. Then, hierarchical clustering was carried out to pinpoint gene modules. Modules were color-coded for visualization. We correlated module eigengenes (MEs) with ssGSEA scores for glycolysis and ketone metabolism pathways. Screening criteria for trait-associated modules were defined as |r| > 0.3 and *p* < 0.05. The module exhibiting the highest correlation with both phenotypes (glycolysis and ketone metabolism) was selected for downstream analysis. This module comprised 658 genes. Key genes linked to glycolysis and ketone metabolism were identified using thresholds of module membership (MM) > 0.8 and gene significance (GS) > 0.2, yielding 54 genes associated with the glycolysis- and ketone metabolism-related module. An intersection was taken between the 761 DEGs and the 54 genes related to glycolysis and ketone metabolism-related modules, and the resulting genes were denoted as candidate genes.

### 2.4 Functional gene network construction

To further investigate the functional roles of candidate genes, we employed TissueNexus (https://www.diseaselinks.com/TissueNexus/) (20), a database encompassing functional gene networks (FGNs) across 49 human tissues and cell lines. A cardiac tissue-specific FGN subnetwork was constructed by integrating the first-degree neighboring genes of the five candidate genes. Core genes within this subnetwork were subsequently defined as nodes with a network degree > 20.

### 2.5 GO and KEGG analysis

Gene ontology (GO) and Kyoto Encyclopedia of Genes and Genomes (KEGG) pathway enrichment analyses of genes include in cardiac tissue-specific FGN subnetwork of the five candidate genes were executed via “clusterProfiler” package in R software. Statistical significance for enrichment analyses was established at a threshold of *p* < 0.05.

### 2.6 Machine learning analysis

To further obtain the hub gene in the five candidate genes, we utilized the least absolute shrinkage and selection operator (LASSO), which represents a widely utilized regularization technique for high-dimensional prediction modeling, and machine learning algorithms Boruta to select the most crucial gene. The LASSO regression and the Boruta analysis was accomplished using glmnet package and Boruta package in R language, respectively. The candidate gene co-screened by Lasso and Boruta was used as hub gene for subsequent research.

### 2.7 Single-gene GSEA enrichment analysis

We performed systematic functional enrichment analyses using control and heart failure samples from GSE5406、GSE236374 dataset. Gene sets of the glycolysis-related gene sets (GOBP_GLYCOLYTIC_PROCESS_THROUGH_FRUCTOSE_6_PHOSPHATE and GOBP_GLYCOLYTIC_PROCESS_THROUGH_GLUCOSE_6_PHOSPHATE) and ketone metabolism-related gene set (GOBP_CELLULAR_KETONE_METABOLIC_PROCESS) were downloaded from the Molecular Signatures Database (MSigDB, Homo sapiens). Spearman's rank correlation coefficients were computed between hub gene expression and all interrogated genes across samples. These correlation metrics were subsequently employed as ranking criteria for single-sample gene set enrichment analysis (GSEA) using the clusterProfiler package in R. This approach enabled identification of biological pathways significantly enriched (false discovery rate < 0.25, *p* < 0.05) among biomarker candidates exhibiting coordinated expression patterns with hub gene.

### 2.8 Immune infiltration and differentially expressed immune factors analysis

To systematically characterize immune infiltration patterns, single-sample Gene Set Enrichment Analysis (ssGSEA) on transcriptomic datasets stratified into control and heart failure samples were utilized. A predefined gene signature panel encompassing 28 immune cell types derived from the TISIDB database (http://cis.hku.hk/TISIDB/download.php) (21). Gene expression profiles were subjected to immune cell quantification using the GSVA package in R, which estimated enrichment scores for 28 different immune cell populations. The proportion of immune cell subsets across samples was visualized through unsupervised hierarchical clustering heatmaps generated by the pheatmap R package. We performed Spearman correlation analysis between hub gene and differentially expressed immune cells using the R package “psych” in all samples. For differentially expressed immune factors analysis, we conducted comparative analyses of immunomodulatory factor expression between control and heart failure samples within the dataset. Utilizing the Wilcoxon rank-sum test, we evaluated 24 immunosuppressive agents, 45 immunostimulators, and 41 chemokines from previous study for differential expression (*p* < 0.05) (22). Immune factors demonstrating statistically significant inter-group differences were designated as differentially expressed immune factors (DEIFs). The network diagram showing the interaction relationships among hub gene, differentially expressed immune factors, and differentially expressed immune cells was drawn using Cytoscape software.

### 2.9 Hub gene-centered regulatory network establishment

Potential microRNAs (miRNAs) targeting hub gene were predicted using three complementary databases: miRWALK (http://mirwalk.umm.uni-heidelberg.de) (23), miRDB (http://www.mirdb.org) (24), and TargetScan (http://www.targetscan.org) (25). High-confidence miRNAs were defined as those consistently identified across all three platforms, with intersection analysis performed via the VennDiagram package in R. Concurrently, transcription factors (TFs) governing hub gene were acquired from the KnockTF database (http://www.licpathway.net/KnockTF/index.html) (26). The integrated miRNA-TF regulatory network of TIMP1 were obtained from the miRNet tool (https://www.mirnet.ca/) and visualized using Cytoscape software (v3.9.1) (27).

### 2.10 Construction of mouse myocardial infarction model

Male C57BL/6J mice (8 weeks old, SPF grade, *n* = 15) were anesthetized with pentobarbital sodium, followed by orotracheal intubation and mechanical ventilation (120 breaths/min, tidal volume 4 mL). Once stable anesthesia was verified, a left thoracotomy was conducted at the fourth intercostal space, facilitating the exposure of the heart. The left anterior descending (LAD) coronary artery was ligated 3 mm distal to the aortic root using an 8-0 suture, with successful occlusion confirmed by ST-segment elevation on ECG and pallor/hypokinesis of the anterior left ventricular wall. The thoracic cavity was closed in layers, and pneumothorax was prevented via syringe aspiration. Postoperatively, mice were monitored for respiratory recovery and housed individually with free access to food/water. Hearts were harvested 7 days post-MI for further analysis. Key reagents and equipment included ophthalmic surgical tools, a rodent ventilator (RWD HF-12), and standard disinfectants (75% alcohol, saline).

### 2.11 Mouse ultrasound imaging experiment

Prior to echocardiography, the ultrasound probe was connected to the instrument interface, and the animal handling platform was preheated. Mice were anesthetized with 2% isoflurane and securely positioned on the scanning platform, with physiological monitoring signals (e.g., ECG, respiration) established. B-mode imaging was performed in both long- and short-axis views, and raw data were saved in animal-specific folders. Cardiac chamber dimensions and function were analyzed offline using the manufacturer's software by manually tracing endocardial borders at end-systolic and end-diastolic phases. Operators monitored all procedures pre- and post-experiment to ensure data objectivity.

### 2.12 RNA extraction and qPCR analysis of mouse cardiac tissue

After sacrificing anesthetized mice, excise the heart, rinse with ice-cold PBS, blot dry, grind 50–100 mg tissue to powder in liquid nitrogen, transfer to a tube with 1 ml TRIzol, Using a vortex to lyse cells, incubate the lysate, add chloroform, centrifuge the mixture, transfer the aqueous phase, precipitate RNA with isopropanol, wash the precipitate with 75% ethanol, air-dry the RNA, dissolve it in RNase-free water, quantify the RNA using NanoDrop, and store the RNA at −80°C. For qPCR, use a TaKaRa reverse transcription kit and Roche SYBR Green Master Mix on a Bio-Rad CFX96 machine: design primers (e.g., GAPDH as control) with Primer3 and BLAST, reverse transcribe 1 μg RNA with random hexamers, dNTPs, buffer, RNase inhibitor, and reverse transcriptase, then set up 20 μl reactions with cDNA, primers, and master mix, performing 40 cycles (95°C denaturation, 60°C annealing, 72°C extension) and mRNA expression of target genes was normalized to GAPDH via the 2^(-ΔΔCt) method with statistical tests like *t*-tests or ANOVA. All qPCR experiments were performed in triplicate. Primers used in this study were as follows:

TIMP1-F:CAGTGTTTCCCTGTTTATCTATCCC.

TIMP1-R: GCAAAGTGACGGCTCTGGTAG.

THBS4-F: GGTCTTTGATCTTCTACCGTCCTC.

THBS4-R: AAGGTGGAGATGAGATAGACTTCGTG.

HCLS1-F: GTTGGGGAGTTAGATCGGCA.

HCLS1-R:GGTCCAGCTTGGTAGGACAG.

C5AR1-F:GCAGCCCTTATCATCTACTCGG.

C5AR1-R: CCGCCAGATTCAGAAACCAG.

GAPDH-F: CCTCGTCCCGTAGACAAAATG.

GAPDH-R: TGAGGTCAATGAAGGGGTCGT.

## 3 Results

### 3.1 Screening of differentially expressed genes in heart failure

**Figure 1:**
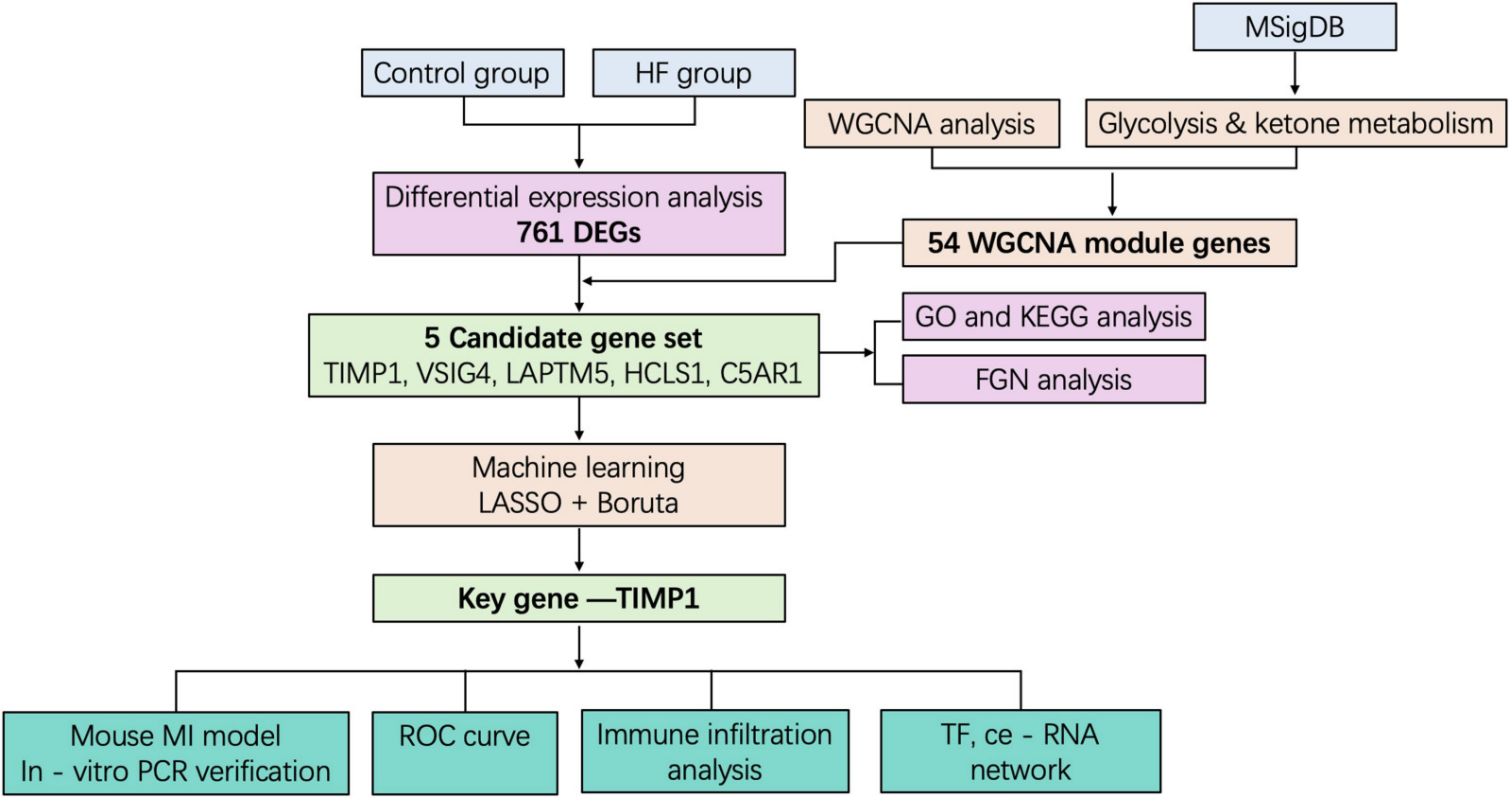
The flowchart of this study.

**Figure 2:**
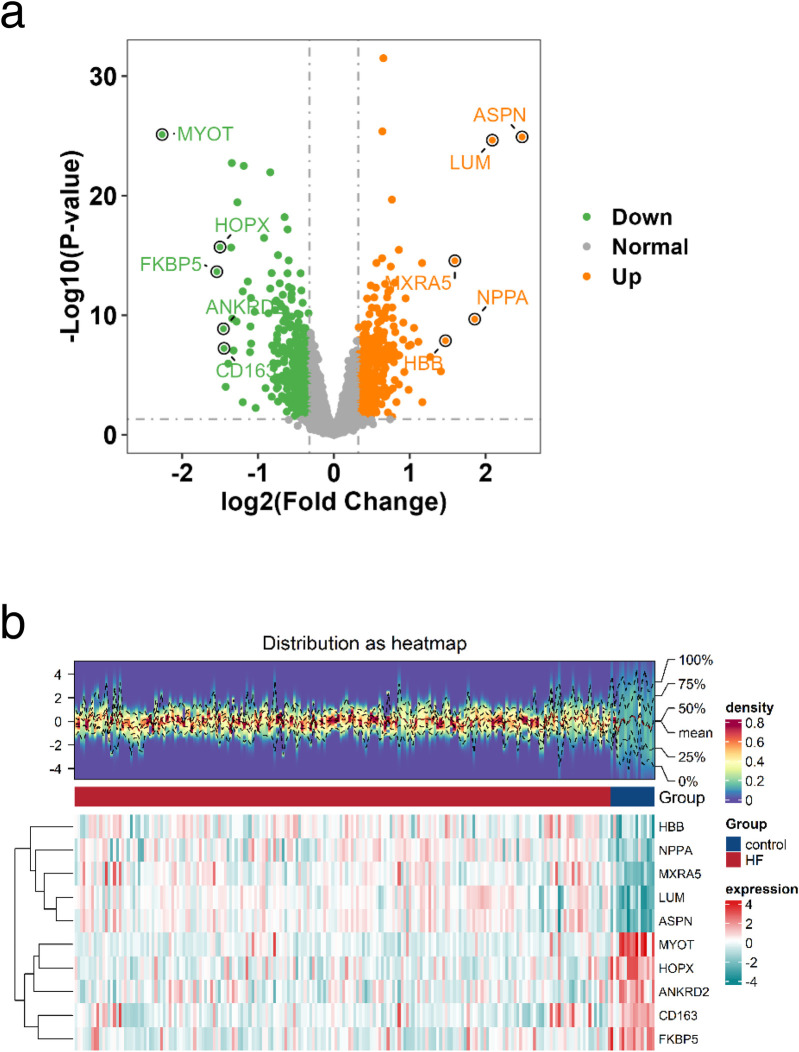
Identification of DEGs. **a** Volcano plot of the distribution of DEGs, Orange indicates upregulated genes; green indicates downregulated genes and grey indicates genes excluded by DEG screening criteria. **b** Heatmap of DEGs, The upper part is a density heatmap of expression levels for upregulated and downregulated genes in samples, showing lines of five quantiles and the mean. The lower part has each column representing a sample. This figure plots the top 5 upregulated genes and top 5 downregulated genes sorted by the log2FC fold change. Each row represents a gene, displaying its expression levels across different samples. The color of the heatmap indicates the magnitude of gene expression in samples: the higher the expression, the darker the color (red for high expression, blue for low expression).

To investigate the characteristic genes of glycolysis and ketone body metabolism in the occurrence and development of heart failure (HF), we designed this study as outlined in Figure 1. First, to identify genes related to HF, we downloaded the transcriptome sequencing dataset GSE5406 from the GEO database, which encompassed 16 control left ventricular hearth tissue samples and 194 HF tissue samples. Differential expression analysis was executed between the control and HF groups. In this study, genes with *p*-value < 0.05 and fold change > 1.25 were defined as up-regulated genes in HF, while those with *p*-value < 0.05 and fold change < 1/1.25 were defined as down-regulated genes in HF. Among them, there were 373 down-regulated genes and 388 up-regulated genes (Supplementary Table S1). The expression distribution of the top 10 genes with the largest fold change is shown in Figure 2a. Heatmaps further illustrated the top five upregulated (HBB, NPPA, MXRA5, LUM, ASPN) and downregulated (MYOT, HOPX, ANKRD2, CD163, FKBP5) genes in HF (Figure 2b).

### 3.2 Identification of glycolysis- and ketone metabolism-related gene modules

**Figure 3:**
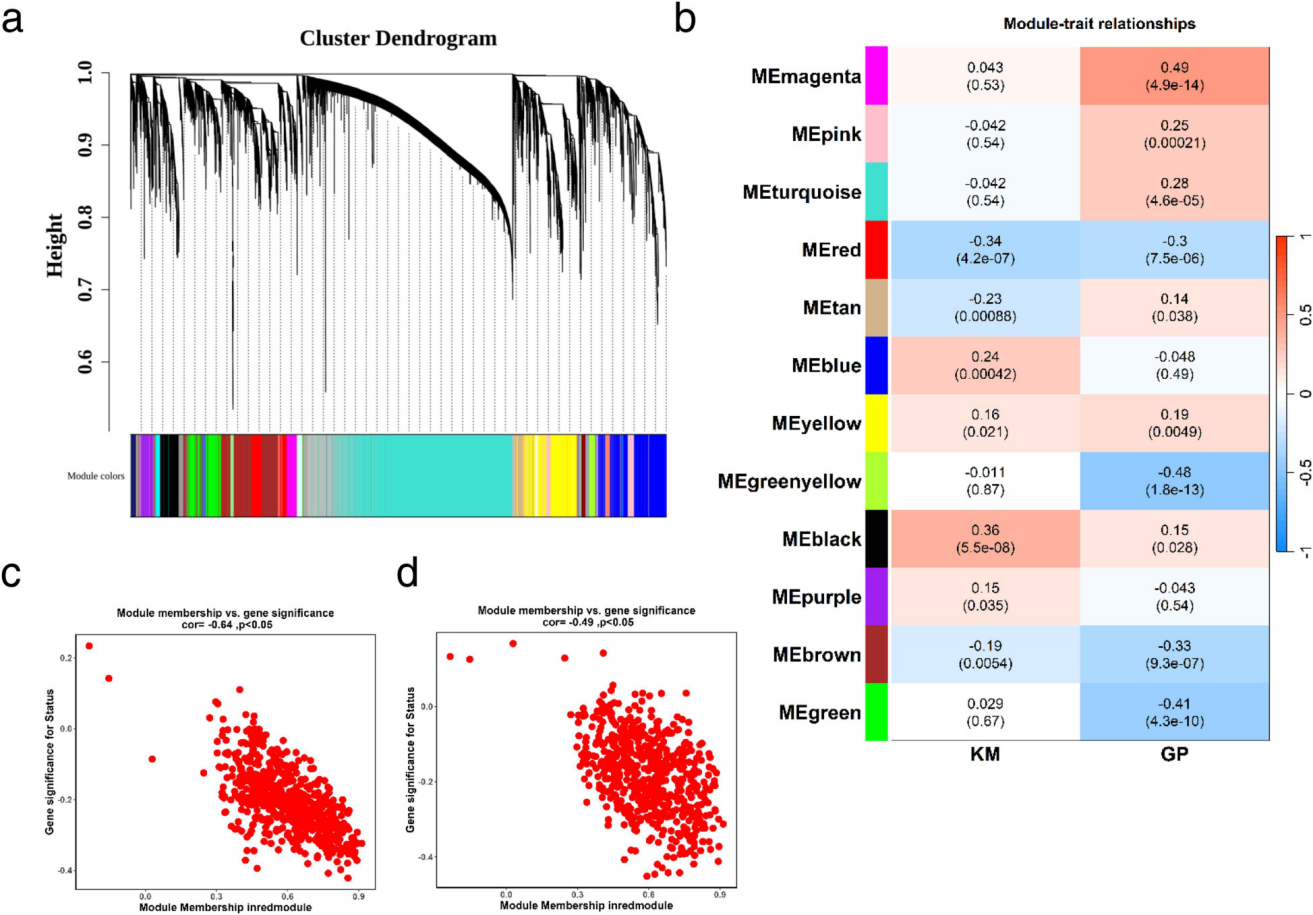
Identification of glycolysis- and ketone metabolism-related gene modules. **(a)** Identification of co-expression modules: the upper part is a hierarchical clustering dendrogram of genes, and the lower part shows gene modules. The upper and lower parts correspond to each other, where genes with closer distances (clustered into the same branch) are assigned to the same module. **(b)** Correlation heatmap between modules and phenotypes: the color blocks on the far left represent modules, and the color bar on the far right indicates the correlation range. In the central heatmap, darker colors signify higher correlation; red denotes positive correlation, and blue denotes negative correlation. The numbers in each cell represent the correlation coefficient and significance. **(c,d)** Correlation between key module genes and ketone metabolism **(c)** or glycolysis **(d)**. MM represents the correlation between genes and modules, and GS represents the correlation between genes and traits.

Weighted gene co-expression network analysis (WGCNA) is designed to identify co-expressed gene modules, explore the relationship between gene networks and target traits, and screen out key gene modules of interest. First, all samples were clustered, hierarchical clustering was performed using the Euclidean distance of expression levels, outliers in the samples were checked, and outlier samples were excluded. As shown in Supplementary Figure S1a, no outlier samples were excluded in this study. A suitable soft threshold power was selected from 1 to 20 to determine the threshold of gene correlation. The relationship between the soft threshold *β* and the scale-free network evaluation coefficient R^2^, as well as the relationship between the soft threshold power and the average connectivity, were established. As shown in Supplementary Figure S1b, set R^2^ = 0.85, and we screened the soft threshold exceeding the red cutting line. As shown in Supplementary Figure S1c, we screened the soft threshold with connectivity close to 0. Finally, a soft thresholding power (*β* = 7) was selected to guarantee scale-free network topology. Twelve co-expression modules were identified (excluding the grey module to which genes that could not be classified belonged, Figure 3a). We took the glycolysis and ketone metabolism gene sets from the MSigDB database and further used the ssGSEA scores of glycolysis and ketone metabolism as phenotypes to construct the association between phenotypes and modules, calculated the correlation coefficient matrix between module eigenvectors and phenotypic traits, and then generated a correlation heat map to visualize the results. As shown in Figure 3b, the red module showed significant correlations with both phenotypes (|r| > 0.3, *p* < 0.05). Then, the correlation between module genes and traits was drawn separately. The red module had 658 genes, and then we screened 54 key genes of the red module through the criteria: mm > 0.8 and gs > 0.2, which were used for subsequent analysis (Figure 3c and Supplementary Table S1).

### 3.3 Identification of glycolysis/ketone metabolism-related DEGs and functional enrichment

**Figure 4:**
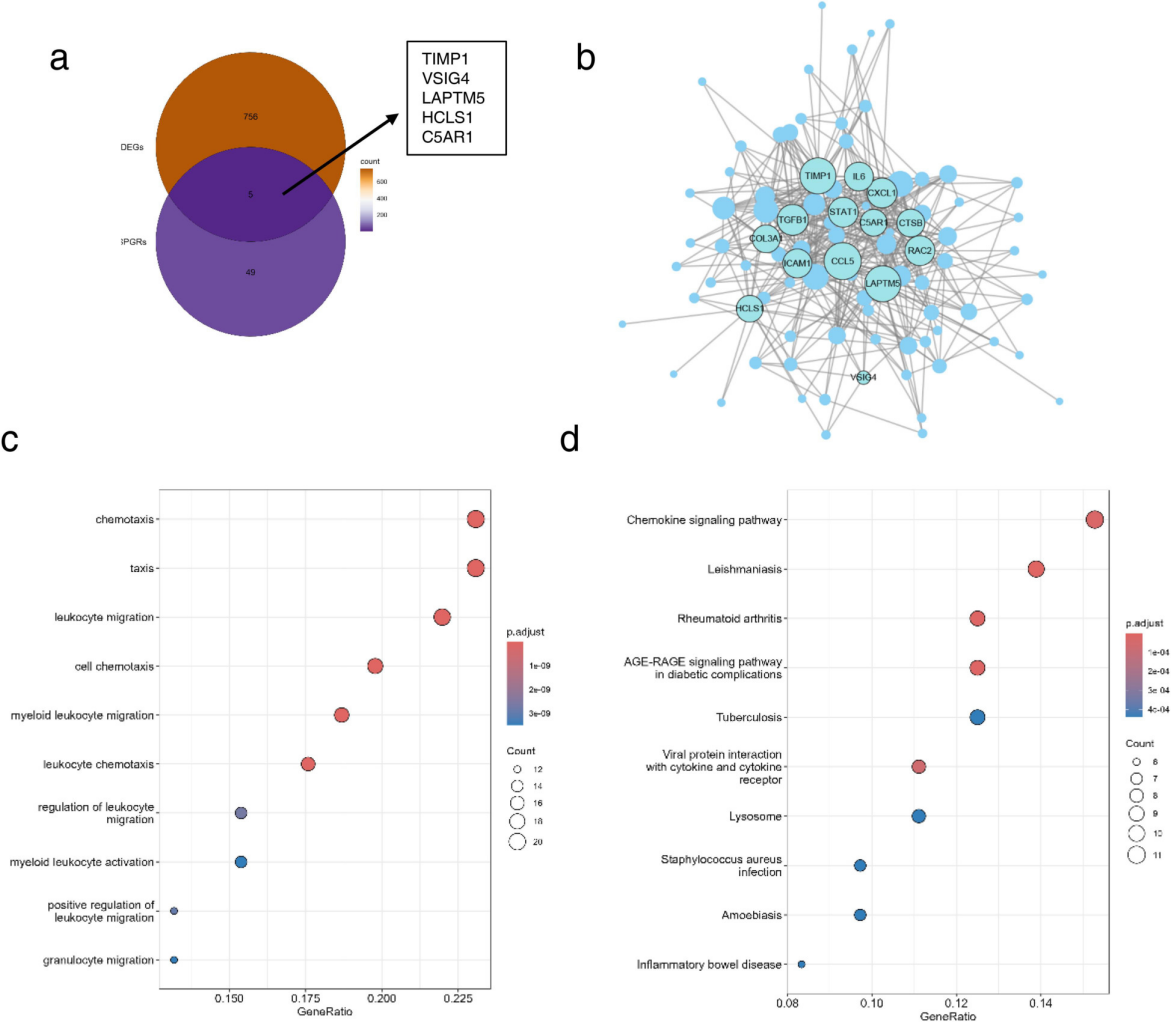
Identification of candidate genes and functional enrichment. **(a)** Venn diagram of DEGs and glycolysis- and ketone metabolism-related genes. **(b)** Subnetwork of cardiac tissue FGNs, the node size represents the degree of the node, and core nodes with degree >20 in the network are labeled. **(c,d)** Functional enrichment **(c)** and pathway enrichment **(d)** of the subnetwork of cardiac tissue FGNs. The node size in the figure represents the number of genes overlapping between the nodes in the network and the gene set with enriched functions.

Through the above steps, we obtained 761 DEGs between HF and control groups, and 54 glycolysis- and ketone metabolism-related module genes. We intersected these two gene sets, and the overlapping genes were regarded as glycolysis- and ketone metabolism-related DEGs, which are hereafter referred to as candidate genes for simplicity. It yielded a total of 5 candidate genes, namely TIMP1, VSIG4, LAPTM5, HCLS1, and C5AR1 (Figure 4a). We then constructed functional regulatory networks (FGNs) for the 5 candidate genes through TissueNexus. We used the one-step neighbors of the candidate genes to obtain their sub-network in the cardiac tissue FGNs and labeled the core genes (degree > 20) in this sub-network. We found that TIMP1, LAPTM5, HCLS1, and C5AR1 among the candidate genes were in core positions in the FGN of cardiac tissue (Figure 4b). We continued to carry out GO functional enrichment on the above cardiac tissue FGNs, and the results highlighted processes such as cell chemotaxis and leukocyte migration (Figure 4c), while KEGG analysis identified chemokine signaling as a key pathway (Figure 4d).

### 3.4 Machine learning identifies key genes in HF glycolysis and ketone metabolism regulation

**Figure 5:**
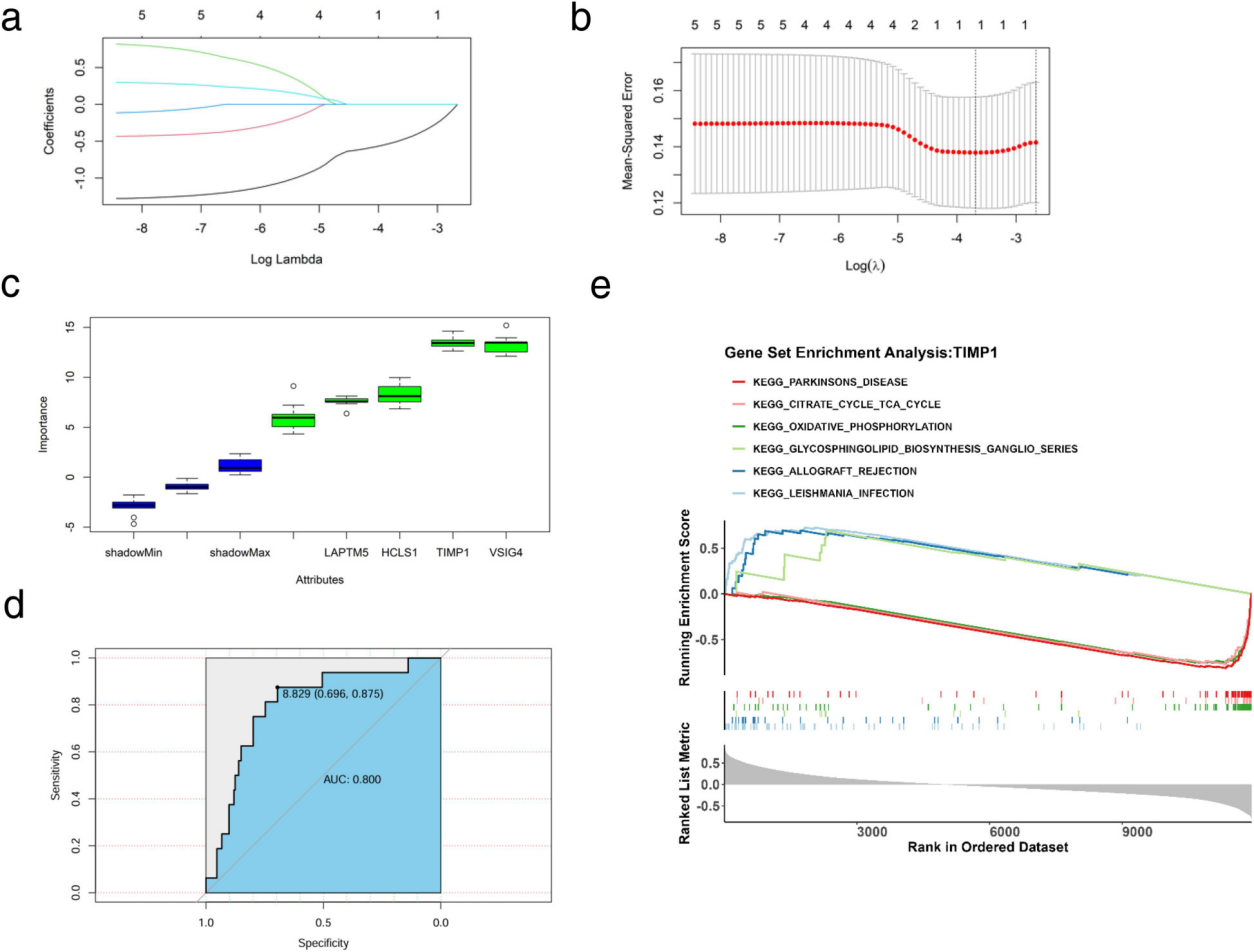
Machine learning and key gene identification. **(a)** Coefficient spectrum diagram of the Lasso model construction, with the abscissa as the logarithm of lambdas and the ordinate as the variable coefficient. When the optimal lambda is reached, variables with coefficients equal to 0 are eliminated. **(b)** Ten-fold cross-validation for adjusting parameters in the Lasso model construction, with the abscissa as the logarithm of lambdas and the ordinate as the model error. The optimal lambda value is at the lowest point of the red curve, corresponding to 1 variable. **(c)** Importance of candidate genes for diagnostic accuracy. (d) ROC curve of TIMP1 for HF diagnosis. **(e)** Single-gene enrichment analysis of TIMP1, the upper part represents the functional score curve, the middle part represents genes in functional pathways, and the lower part represents functional correlations.

To further obtain key genes regulating HF via glycolysis and ketone metabolism, we leveraged machine learning algorithms to narrow down the candidate gene range. Specifically, we first applied LASSO regression to construct a penalized function for model refinement. After performing Lasso regression analysis on 5 candidate genes, we found that the optimal lambdamin value was 0.025 (Figure 5a), and finally, a total of one gene whose regression coefficient was not penalized to 0 was obtained, namely TIMP1 (Figure 5b). Besides, Boruta was implied to calculate the importance of the features, as shown in Figure 5c, all candidate genes were accepted by Boruta. We finally decided that the candidate genes jointly screened by Lasso and Boruta were used as the key genes for subsequent research, namely TIMP1. We drew the ROC curve of TIMP1, in which AUC = 0.80, indicating that the expression of TIMP1 has a diagnostic potential on HF (Figure 5d). To identify the biological functions involved in TIMP1, we used the “c2.cp.kegg.v7.0.symbols.gmt” in the MSigDB database as the reference gene set, performed Spearman correlation analysis between TIMP1 and all genes, and obtained the correlation coefficient. Taking the correlation coefficient as the sorting standard, we carried out single-gene GSEA enrichment analysis on TIMP1. The results showed that TIMP1 was enriched in pathways including allograft rejection, glycosphingolipid biosynthesis ganglion series, citric acid cycle recycling, and Parkinson's disease (Figure 5e).

### 3.5 Immune regulatory network of key gene TIMP1

**Figure 6:**
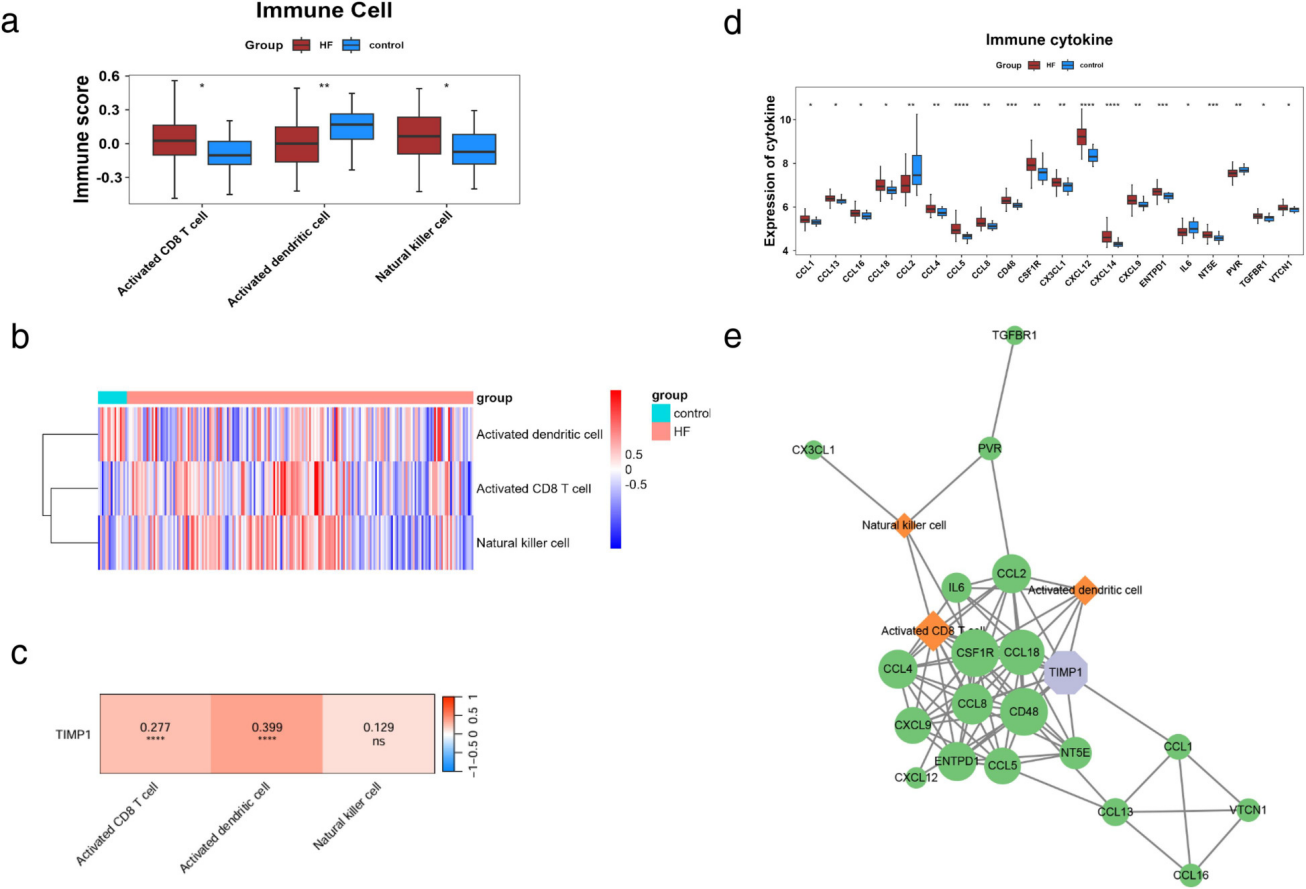
Immune regulatory network of TIMP1. **(a)** Differential immune cell scores. **(b)** Distribution of differential immune cell scores, the upper color represents sample information, and the lower color represents immune cell scores. Blue indicates low scores, and red indicates high scores. **(c)** Correlation heatmap between TIMP1 and differential immune cells, colors represent the strength of correlation, where red indicates positive correlation, blue indicates negative correlation, "ns" denotes non-significant correlation, and "*" denotes significant correlation. **(d)** Differential immune cytokine scores. **(e)** Immune network of TIMP1,green represents differential immune factors, orange represents differential immune cells, purple represents GK characteristic genes, and edges in the network represent correlations > 0.25.

To explore the role of immune cells in HF, we conducted immune infiltration analysis on the control and HF groups using the ssGSEA algorithm. We obtained gene sets for 28 immune cell types from the TISIDB database and calculated the enrichment scores of immune infiltrating cells in each sample. The results indicated 3 differentially abundant immune cells in HF: activated CD8 T cell, activated dendritic cell, and natural killer cell (Figures 6a,b). To further understand the connection between TIMP1 expression and differential immune cells, we performed Spearman correlation analysis between the expression of TIMP1 and the above 3 differential immune cells. We found that two differential immune cells were significantly positively correlated with the expression of TIMP1, including activated CD8T cell and activated dendritic cell (Figure 6c), indicating that the expression change of TIMP1 is related to the alteration of the immune microenvironment in HF. We further evaluated the significance of immune factors in HF. We obtained the immune factor gene list reported in previous studies including 24 immune inhibitors, 45 immune stimulants, and 41 chemokines (2), and used the Wilcoxon test in our dataset to compare the expression level differences of immune factors between the control group and the HF group (*p* < 0.05). The immune factors with significant differences were defined as “differential immune factors”. We screened a total of 20 differential immune factors (Figure 6d). We used Cytoscape to draw a network diagram based on TIMP1, differential immune factors, and differential immune cells to show the interaction relationship between TIMP1, differential immune factors, and differential immune cells. It is presented that TIMP1 could be associated with differential immune cells through a variety of differential immune factors. For example, TIMP1 could be linked to activated dendritic cell via CCL2 (Figure 6e).

### 3.6 Transcriptional regulation and validation expression of candidate genes in HF models

**Figure 7:**
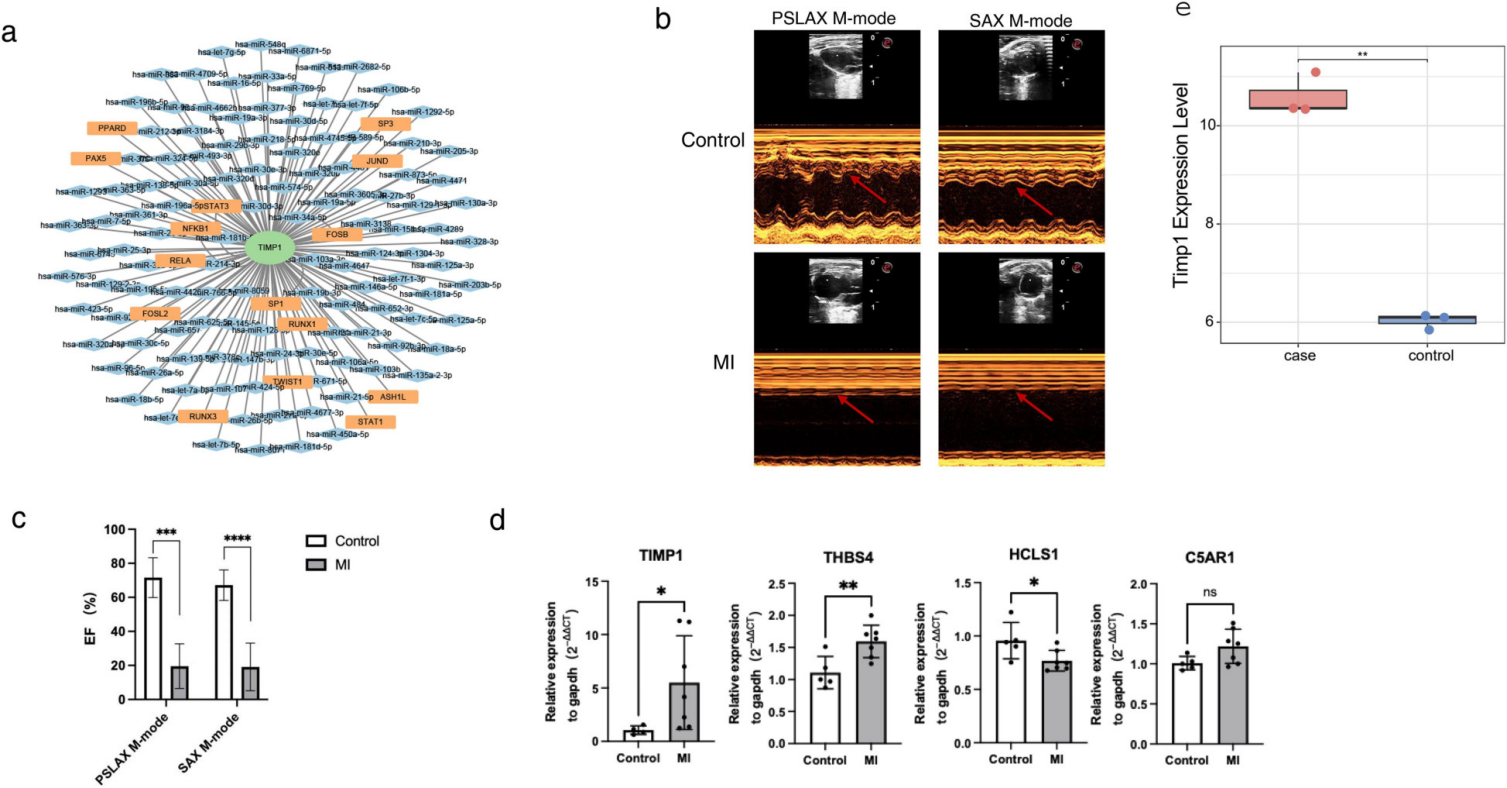
Transcriptional regulatory network of TIMP1 and in vitro model validation. **(a)** miRNA-TF regulatory network of TIMP1. Green nodes represent biomarkers, orange nodes represent TFs (transcription factors), blue nodes represent miRNAs, and edges in the network represent regulatory relationships. **(b)** Cardiac ultrasound images of myocardial infarction mice and control mice. The arrow indicates the morphology of the M-wave. **(c)** Statistical results of cardiac ejection fraction in myocardial infarction mice and control mice. **(d)** qPCR detection of gene expression in cardiac tissues of myocardial infarction mice and control mice. **(e)** Expression of TIMP1 in GSE236374. * represents p < 0.05, ** represents *p* < 0.01, *** represents *p* < 0.001, **** represents *p*<0.0001.

To explore the molecular regulatory mechanism of TIMP1, we also explored the potential transcriptional regulation modes upstream of TIMP1. miRNA and transcription factors (TF) can play a role in maintaining physiological stability by regulating the expression of target genes. Therefore, we used the miRNet database to obtain the upstream miRNA-TF regulatory network of TIMP1 and used Cytoscape for visualization. miRNet analysis predicted 135 upstream regulators of TIMP1 (15 TFs and 120 miRNAs; Figure 7a). To verify the importance of candidate genes in heart failure, we constructed a mouse myocardial infarction (MI) model. The results of mouse cardiac ultrasound showed that the M wave in the MI group was obviously changed to a straight line (Figure 7b), and the ejection fraction of the heart in the MI group was significantly lower than that in the control group (Figure 7c). The above results showed that our model was successfully constructed. Seven days after MI in mice, we took the heart tissue of mice, extracted RNA, and detected the expression of several red module genes by qPCR. For qPCR validation, we selected genes representing different functional categories within the WGCNA red module: (1) TIMP1—the hub gene identified by machine learning; (2) HCLS1 and C5AR1—two of the five candidate genes with known immune regulatory functions; (3) THBS4 (Thrombospondin 4)—although not among the final five candidate genes, THBS4 was a highly significant DEG (log2FC = 8.36, *p* < 0.0001) within the red module with established roles in extracellular matrix remodeling and cardiac fibrosis (28). THBS4 was included as a positive control to validate the broader WGCNA module's relevance to HF pathology.The results are shown in Figure 7d. In heart tissue samples, we observed that TIMP1 and THBS4 expression levels were significantly upregulated in HF group compared to the control group. Conversely, HCLS1 expression was notably downregulated in the HF group, while C5AR1 expression showed no significant difference between the two groups. We found that there was partial consistency between animal modeling qPCR results and GES5406 sequencing data. For example, THBS4 and HCLS1 were up-regulated and down-regulated genes in the dataset, respectively. However, TIMP1 was highly expressed in the HF group in qPCR, but TIMP1 belonged to the down-regulated gene in the HF group in the GES5406 dataset. We speculated that this might be due to the fact that the dataset was from human samples, while our verification was in mouse tissues. In response to this concern, we have analyzed an additional mouse heart failure dataset, GSE236374, which utilizes the same animal modeling approach as our study. Our expression analysis demonstrated that TIMP1 expression was significantly elevated in the acute myocardial infarction group, which is in agreement with the findings from our animal model (Figure 7e). We guessed that the mechanism of action of TIMP1 in HF might have species differences. Species-specific discrepancies (e.g., TIMP1 expression trends) suggest divergent regulatory mechanisms between human and mouse HF.

## 4 Discussion

This study focuses on HF—a primary driver of global cardiovascular mortality—and systematically elucidates the cascade mechanism by which dysregulated glycolysis/ketone body metabolism drives metabolic-immune microenvironment imbalance through the core hub gene TIMP1, integrating bioinformatics, machine learning, and experimental validation.

This study identified a red module highly correlated with glycolysis/ketone body metabolism through WGCNA. Its 54 core genes predominantly drive the metabolic reprogramming in heart failure (characterized by a shift from fatty acid oxidation towards glycolysis/ketone utilization). Key candidate genes include: TIMP1: Suppresses MMPs, regulating fibrosis and inflammation (29, 30). LAPTM5: Drives TNF-α/IL-1β secretion from macrophages via the lysosomal-TLR signaling pathway (31). HCLS1: Mediates leukocyte migration (32). C5AR1: Activates neutrophil chemotaxis (33). VSIG4: Modulates macrophage immunotolerance (34). Functional Gene Networks (FGNs) positioned TIMP1, LAPTM5, HCLS1, and C5AR1 as core nodes. GO and KEGG enrichment analyses confirmed their significant association with leukocyte chemotaxis, regulation of cell migration, and the chemokine signaling pathway. These genes synergistically drive HF progression through a three-pronged mechanism: C5AR1/HCLS1-mediated immune cell infiltration amplifies inflammation (35, 36); TIMP1-mediated MMP inhibition accelerates fibrotic remodeling; LAPTM5-promoted TNF-α/IL-1β secretion suppresses mitochondrial function, forcing a reliance on glycolytic energy production. In summary, the red module genes, particularly the core candidates, directly link glycolytic/ketone metabolic imbalance to immune microenvironment dysregulation in HF (37), by regulating chemokine signaling-mediated leukocyte migration and activation (38). Collectively, they promote myocardial remodeling and functional deterioration.

While TIMP1 is classically recognized as a tissue inhibitor of metalloproteinases, emerging evidence demonstrates its pleiotropic functions as a cytokine-like molecule with direct immunomodulatory properties (39). Our findings that TIMP1 expression positively correlates with activated CD8^+^ T cells and dendritic cells are consistent with its established role in immune cell regulation. A critical function of TIMP1 is the direct modulation of macrophage polarization. Evidence suggests its expression is associated with a pro-inflammatory M1 phenotype, though it can also promote anti-inflammatory M2 polarization in certain contexts via MAPK and PI3 K/AKT signaling (40, 41). This indicates TIMP1 has context-dependent, and potentially biphasic, roles during cardiac injury and repair. Furthermore, TIMP1 correlates with various immune cell markers, particularly those of tumor-associated macrophages (42), suggesting its broader role in orchestrating the immune microenvironment. In our study, the strong association between TIMP1 and differential immune factors including CCL2 supports a model where TIMP1 not only responds to but actively shapes the inflammatory milieu in HF. This is consistent with TIMP1's capacity to function through multiple cell surface receptors beyond its MMP-inhibitory function, thereby exerting direct signaling effects on immune cells.

Mechanistically, the link between TIMP1-driven immune activation and metabolic dysfunction relies on evidence-based inferences. Inflammatory mediators like TNF-α and IL-1β—often elevated alongside TIMP1—directly impair mitochondrial function in cardiomyocytes. They inhibit key metabolic regulators such as pyruvate dehydrogenase (PDH) (43) and PGC-1*α* (44), shifting energy production from efficient fatty acid oxidation to a compensatory, increased reliance on glycolysis. This explains the co-enrichment of TIMP1 with both immune and glycolytic pathways observed in our study.

In the context of HF, this creates a vicious cycle: initial cardiac injury → metabolic stress → immune cell infiltration (as evidenced by our finding of increased activated CD8^+^ T cells and dendritic cells) → inflammatory cytokine secretion → further metabolic impairment → chronic low-grade inflammation (45)→ progressive cardiac dysfunction. TIMP1 appears to sit at a critical node within this cycle, both responding to and amplifying both metabolic and immune dysfunction. Collectively, these findings support a model where TIMP1 serves as a central integrator of metabolic stress signals and immune responses in HF, rather than functioning solely as a downstream marker of either process.

Through dual screening via LASSO regression and the Boruta machine learning algorithm, TIMP1 was established as a key diagnostic biomarker for HF. Its expression level effectively distinguishes HF samples from controls. The ssGSEA revealed that TIMP1 expression was significantly enriched in pathways such as “ allograft rejection” and the “ citric acid cycle recycling”. This enrichment suggests TIMP1 drives HF progression through a dual mechanism: Mediating inflammatory responses (46), and Suppressing mitochondrial energy metabolism (47). Immune infiltration analysis further confirmed that TIMP1 expression exhibited a significant positive correlation with the infiltration of activated CD8+ T cells and dendritic cells. Mechanistically, this may occur through TIMP1-induced expression of chemokines like CCL2 (48), recruiting immune cells to myocardial tissue. This recruitment directly drives inflammatory injury and synergizes with metabolic dysregulation to accelerate fibrotic processes (49). Collectively, these findings support the translational value of TIMP1 as both a novel diagnostic biomarker for HF and a dual metabolic-immune regulatory therapeutic target.

The ssGSEA initially revealed significant enrichment of activated CD8+ T cells, activated dendritic cells, and natural killer (NK) cells in heart failure. Furthermore, subsequent analysis demonstrated that TIMP1 expression exhibited a strong positive correlation specifically with the infiltration levels of activated CD8+ T cells and activated dendritic cells. Building upon these findings and combined with the screening of 20 differential immune factors and Cytoscape-based network construction, it was subsequently confirmed that TIMP1 directly regulates the infiltration of these specific immune cells through chemokines such as CCL2. Importantly, validation in a murine myocardial infarction (MI) model further corroborated these observations, demonstrating significant upregulation of TIMP1 and THBS4 mRNA expression, alongside downregulation of HCLS1. Our miRNet analysis predicted 135 upstream regulators of TIMP1 (15 transcription factors and 120 miRNAs), many of which are key mediators of glycolytic/ketone metabolic reprogramming and immune activation, suggesting TIMP1 serves as a downstream integrator of converging metabolic-immune pathways. Among predicted transcription factors, HIF-1*α* emerges as a critical regulator linking metabolic stress to TIMP1 expression. HIF-1*α* directly controls glycolytic enzyme transcription, promoting the shift from oxidative phosphorylation to glycolysis (50), while also regulating immune cell metabolism and inflammatory responses (51). Through TIMP1 regulation, HIF-1*α* may coordinate ECM remodeling with metabolic-immune adaptations during HF progression. Similarly, NF-*κ*B and AP-1, activated by inflammatory cytokines (TNF-α, IL-1β) and metabolic danger signals, drive TIMP1 expression, creating a feed-forward loop: metabolic dysfunction → inflammatory cytokine release → NF-*κ*B/AP-1 activation → TIMP1 upregulation → further immune cell recruitment.

The predicted miRNA regulators provide additional mechanistic links. The miR-29 dysregulation leads to PGC-1*α* suppression, mitochondrial dysfunction, and compensatory glycolysis upregulation (52, 53)—precisely the metabolic phenotype associated with TIMP1 expression. The miR-29/TIMP1 axis thus mechanistically links mitochondrial dysfunction, metabolic reprogramming, and fibrotic remodeling. The convergence of metabolic stress-responsive TFs (HIF-1*α*, NF-*κ*B) and metabolism-regulating miRNAs (miR-29) on TIMP1 suggests it serves as a nodal point integrating diverse metabolic-inflammatory signals. This explains why TIMP1 correlates with both glycolytic/ketone pathway dysregulation and immune cell infiltration in our analysis. Future CRISPR-Cas9 studies targeting these upstream regulators will be essential to establish their causal roles in TIMP1-mediated metabolic-immune dysfunction. Consequently, based on this integrated evidence, we propose the core hypothesis that dysregulation of upstream TF/miRNA networks drives TIMP1 overexpression. This overexpression, in turn, collaboratively promotes HF progression through a triad of mechanisms: namely, CCL2-mediated immune cell infiltration, MMP inhibition-induced fibrosis, and glycolytic/ketone metabolic imbalance. Finally, observed species-specific differences suggest the existence of regulatory heterogeneity within this pathway.

The observed discrepancy in TIMP1 expression patterns—upregulation in our mouse MI model vs. downregulation in human GSE5406 samples—likely reflects temporal dynamics rather than species-specific regulation *per se*. Our mouse model represents acute myocardial injury (7 days post-MI) during the active inflammatory and reparative phase, whereas the GSE5406 dataset comprises end-stage chronic HF samples with established ventricular remodeling. Studies demonstrate that TIMP1 mRNA and protein are significantly upregulated in deteriorating heart failure patients (54) and in cardiac tissue from patients with chronic pressure overload (55). Importantly, TIMP1 is consistently upregulated in myocardial fibrosis during active remodeling phases (56). However, in very advanced end-stage disease with extensive fibrosis and cardiomyocyte loss, cardiac TIMP1 transcription may decline while circulating TIMP1 levels remain elevated due to extracardiac sources (hepatic production, activated immune cells) (57). This interpretation is further supported by recent findings showing that TIMP1 gene transcriptional activity decreases with advancement of heart failure (58), particularly in ischemic cardiomyopathy with severe dysfunction. The temporal trajectory—early upregulation (captured in our acute MI model) followed by late-stage downregulation (reflected in GSE5406 end-stage samples)—aligns with the known biphasic nature of post-infarction remodeling. Therefore, the apparent discrepancy actually reveals disease stage-dependent TIMP1 regulation, emphasizing the importance of considering temporal context when interpreting biomarker expression in HF.

While this study elucidates the TIMP1-mediated metabolic-immune regulatory network in heart failure, several limitations warrant acknowledgment. Firstly, the observed divergent expression trends of TIMP1 across species necessitate the development of humanized models to elucidate the underlying regulatory heterogeneity. Secondly, the causal relationships between the identified key upstream TFs/miRNAs and metabolic pathway dysregulation remain to be functionally validated *in vivo*. Thirdly, the immune infiltration profiles, derived from computational algorithms (ssGSEA), require experimental confirmation (e.g., flow cytometry, immunohistochemistry) to definitively establish the mechanisms of CD8+ T cell and dendritic cell infiltration. Therefore, future research should focus on utilizing CRISPR-Cas9 technology to perform targeted editing of upstream regulatory factors of TIMP1, thereby elucidating their causal impact on metabolic reprogramming. Besides, experiments such as, TIMP1 knockout/overexpression models combined with metabolic flux analysis (seahorse assay, LC-MS metabolomics); Co-culture experiments with cardiomyocytes and immune cells were needed to demonstrate TIMP1-mediated metabolic-immune crosstalk. Concurrently, qPCR/Western blot validation in human cardiac tissue samples or developing human cardiac organoid models will be crucial to dissect the mechanisms governing species-specific TIMP1 expression. Ultimately, evaluating the therapeutic potential of targeting the TIMP1-chemokine axis for reversing metabolic-immune dysregulation represents a critical translational goal. While our study primarily focuses on the diagnostic utility of TIMP1, its elevated expression in HF patients and significant correlation with cardiac remodeling parameters suggest its potential as a therapeutic target. However, comprehensive preclinical validation studies, including mechanistic investigations in cellular and animal models, pharmacological modulation experiments, and long-term efficacy assessments, would be essential before drawing definitive conclusions about its therapeutic applicability.

## 5 Conclusion

This study is the first to establish TIMP1 as the central hub integrating dysregulation of the glycolysis-ketone metabolism axis with immune microenvironment imbalance in heart failure. Through integrated multi-omics analysis and experimental validation, we elucidate a stepwise cascade mechanism: Dysregulation of upstream TF/miRNA networks drives TIMP1 overexpression, which subsequently promotes CCL2-mediated immune cell infiltration. This immune dysregulation, in turn, contributes to metabolic imbalance and ultimately leads to myocardial remodeling. This work provides both a novel therapeutic target and a conceptual framework for developing precision-targeted strategies to modulate the metabolic-immune interface in heart failure.

## Data Availability

The datasets GSE5406, GSE236374 for this study can be found in the Gene Expression Omnibus (GEO) database (https://www.ncbi.nlm.nih.gov/geo/).
