# Supplementary material for: Identification and validation of key biomarkers of the glycolysis-ketone body metabolism in heart failure based on multi-omics and machine learning
Source: Front Cardiovasc Med. 2025 Nov 27;12:1672513. doi: 10.3389/fcvm.2025.1672513 (PMC12695798; doi:10.3389/fcvm.2025.1672513)
Supplement: Supplementary file 2 [file Image1.pdf]

Supplementary Figure 1

a

b c

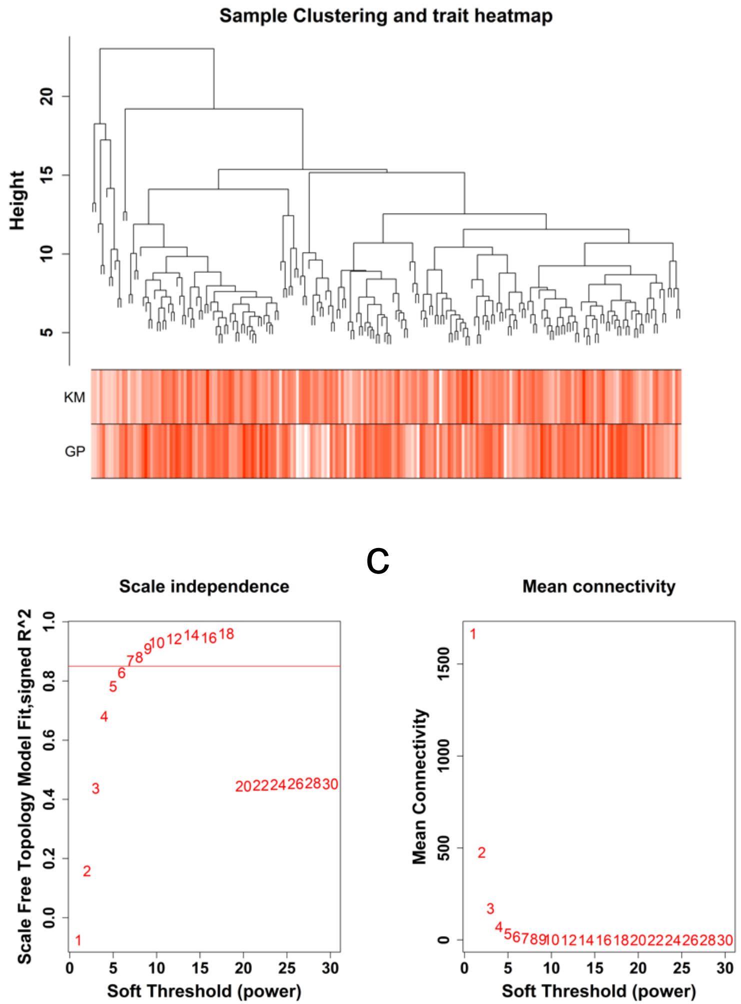

Supplementary Figure 1. Parameter selection for WGCNA analysis. a Hierarchical clustering of samples after introducing sample traits. b-c Soft-threshold screening: Scale-free fit index (y-axis) under different soft-thresholds (x-axis) and network connectivity under different soft-thresholds(c).
